# Evaluation of Artifact Appearance and Burden in Pediatric Brain Tumor MR Imaging with Compressed Sensing in Comparison to Conventional Parallel Imaging Acceleration

**DOI:** 10.3390/jcm12175732

**Published:** 2023-09-03

**Authors:** Rieke Lisa Meister, Michael Groth, Shuo Zhang, Jan-Hendrik Buhk, Jochen Herrmann

**Affiliations:** 1 Department of Diagnostic and Interventional Radiology and Nuclear Medicine, Section of Pediatric Radiology, University Medical Center Hamburg-Eppendorf, 20251 Hamburg, Germany; 2 Department of Medical Imaging, Southland Hospital, Invercargill 9812, New Zealand; 3 Department of Radiology, St. Marienhospital Vechta, 49377 Vechta, Germany; 4 Philips Healthcare, 22335 Hamburg, Germany; zhang.shuo@philips.com; 5 Department of Neuroradiology, Asklepios Kliniken St. Georg und Wandsbek, 22043 Hamburg, Germany

**Keywords:** compressed SENSE, acceleration technique, image quality, brain neoplasms, children

## Abstract

Clinical magnetic resonance imaging (MRI) aims for the highest possible image quality, while balancing the need for acceptable examination time, reasonable signal-to-noise ratio (SNR), and lowest artifact burden. With a recently introduced imaging acceleration technique, compressed sensing, the acquisition speed and image quality of pediatric brain tumor exams can be improved. However, little attention has been paid to its impact on method-related artifacts in pediatric brain MRI. This study assessed the overall artifact burden and artifact appearances in a standardized pediatric brain tumor MRI by comparing conventional parallel imaging acceleration with compressed sensing. This showed that compressed sensing resulted in fewer physiological artifacts in the FLAIR sequence, and a reduction in technical artifacts in the 3D T1 TFE sequences. Only a slight difference was noted in the T2 TSE sequence. A relatively new range of artifacts, which are likely technique-related, was noted in the 3D T1 TFE sequences. In conclusion, by equipping a basic pediatric brain tumor protocol for 3T MRI with compressed sensing, the overall burden of common artifacts can be reduced. However, attention should be paid to novel compressed-sensing-specific artifacts.

## 1. Introduction

Magnetic resonance imaging (MRI) is considered the gold standard for neuro-oncologic brain imaging [1,2]. Recent technical advances in imaging acceleration have shown clear clinical benefits in a reduction of scan times and improvement of image quality [3,4,5]. In children, shorter examination times are particularly desired to keep sedation duration at a minimum, to minimize the exposure time to radiofrequency-induced energy deposition, and to ensure maximum patient compliance [6,7,8,9].

In this context, different imaging strategies have been integrated into various pediatric imaging schemes over the last several years, demonstrating promising results in children [10,11,12,13]. A common approach is the use of compressed sensing (CS) as an imaging acceleration technique based on variable density sampling, sparsifying transformation, and iterative reconstruction [14]. Since CS was made available for clinical use, little attention has been paid to its effects on common image artifacts so far [15], which are known to have a high impact on image quality and diagnostic confidence.

In the process of equipping our brain tumor protocol with compressed sensing, and assessing image quality during and after implementation, we found that there was a noticeable change in the artifact burden and appearance. Recognition of artifacts related to compressed sensing seemed important in order to avoid misinterpretation.

This study aimed to assess the overall artifact burden and artifact appearances in a standardized pediatric brain tumor MRI by comparing conventional parallel imaging acceleration with CS.

## 2. Materials and Methods

Study population: All children with brain tumors who underwent a brain MRI examination with compressed sensing at our institution between October and December 2019 and who had undergone at least one previous examination using the standard protocol without compressed sensing were retrospectively identified. Of 60 patients, 38 were excluded as one of their two protocols had been modified regarding the number of acquired sequences and overall length of the protocol beyond purely introducing CS. The study cohort included 22 patients, aged 2.3–18.8 years at the time of their CS MRI examination [13]. All children had been diagnosed with varying brain tumor entities (mainly astrocytoma, medulloblastoma, and ependymoma) and had undergone different therapeutic pathways at the time of imaging (surgery, radiation therapy, chemotherapy, or multimodality therapy). Five patients below the age of five years were examined under general anesthesia. For details regarding patient data see Supplementary Materials [13].

MRI Protocol: MRI examinations were performed on a 3.0 Tesla whole-body clinical MRI system (Ingenia, software release R5.6, Philips, Best, The Netherlands). A standard 32-channel receiver head coil (Philips) was used. Ear plugs and noise canceling headphones were given to all patients. Foam pads were used to minimize head motion. Some children preferred to listen to music or watch a movie during their examination. All unsedated patients were instructed to keep still during examinations to avoid movement artifacts.

The pediatric brain tumor MR protocol included both unenhanced and contrast enhanced 3D T1-weighted turbo-field-echo (TFE) sequences with similar technical parameters acquired in the sagittal plane with reconstructions in the axial and coronal planes; an axial fluid-attenuated inversion recovery (FLAIR) sequence; and an axial T2-weighted turbo-spin-echo (TSE) sequence [13,16]. Gadolinium was used as an intravenous contrast agent with a dosage of 0.2 mL Gadoteric Acid (Dotagraf^R^, Jenapharm, Germany)/kg body weight, and enhanced 3D T1 TFE sequences were obtained 3 min after contrast injection.

Sensitivity encoding (SENSE) was applied for conventional parallel imaging acceleration and was combined with the CS principle for ‘Compressed SENSE’ acceleration, the latter employing L1 regularization after wavelet sparsifying transformation and iterative reconstruction. Both acceleration techniques (SENSE and CS) were implemented in the vendor software. Imaging parameters for adaptation of the basic MRI brain tumor protocol to compressed sensing were optimized according to visual observation to ensure best diagnostic image quality, the comparative results of which were published elsewhere [13]. Key imaging parameters are given in Table 1 [13].

Image analysis: Two pediatric radiologists, with 16 years (JH) and 13 years (MG) of experience, evaluated artifact burden and strength of artifacts during a consensus reading. Readers were blinded for clinical information and technical parameters. In total, 176 sequences were viewed in random order via Centricity PACS Universal Viewer (GE Web Client Version 6.0, Chicago, IL, USA).

Image artifacts were categorized as either physiology-related (motion, ringing, CSF flow, pulsation/ghosting), physics-related (chemical shift, susceptibility effects), or technique-related [17,18,19]. The latter included acceleration technique (e.g., compressed sensing)-specific artifacts that have been described in the literature [15].

Detailed description of artifact types is given in Supplementary Table S2 [15,17,18,20]. Using a 3-point scale, artifacts were rated according to their strength. The scale incorporated information regarding the amount of regional extension and diagnostic disturbance (0 points, no artifacts; 1 point, light artifacts with most underlying or adjacent structures visible, small or focal appearance, only slight diagnostic impairment; 2 points, strong artifacts, underlying or adjacent structures not clearly visible, extensive or multifocal appearance, substantial impairment of diagnostic assessment). For each of the four sequences and for both acceleration protocols (SENSE vs. CS), artifact frequency and artifact strength were determined. For each artifact type, the artifact frequency and the mean artifact strength were calculated. To assure comparable quantitative image quality between both protocols, separate phantom data-based noise maps were acquired for each of the sequences [21], with comparable measured signal-to-noise-ratio (SNR) values.

Statistical Analysis: Statistical analyses were computed with Excel (Version 16.44, 2020, Microsoft Corporation, Redmond, WA, USA), using a paired Wilcoxon test for numeric variables of artifact strength and summarized artifact strength scores to compare corresponding data sets of each of the sequences for both protocols under the assumption that there was no statistical difference (H0) [11,12,13,22]. A *p*-value < 0.05 was considered statistically significant. Data of scores are given as categorical values with *n* = absolute number of affected scans of all 22 patients including percentage, artifact strength scores given as sum of absolute values with mean ± 1 standard deviation, and summarized artifact strength scores given as mean value ± 1 standard deviation.

## 3. Results

In total, an overall reduction in artifact burden was noted for the compressed sensing (CS) protocol, with the four sequences benefiting to different extents with respect to the various artifacts. Results are summarized in Table 2, Table 3, Table 4 and Table 5. 

**Figure 1:**
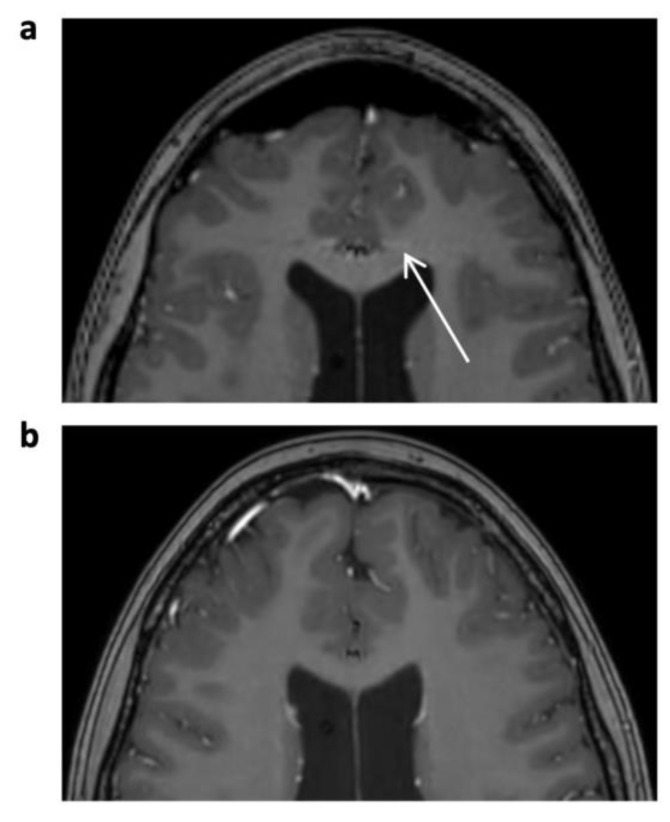
Enhanced 3D T1 TFE images of a 12-year-old male patient with non-germinomatous germ cell tumor (not shown). Pulsation artifact of anterior cerebral artery in phase-encoding direction noted in SENSE 3D T1 TFE ((**a**), arrow); not seen in follow-up imaging with CS 3D T1 TFE (**b**).

**Figure 2:**
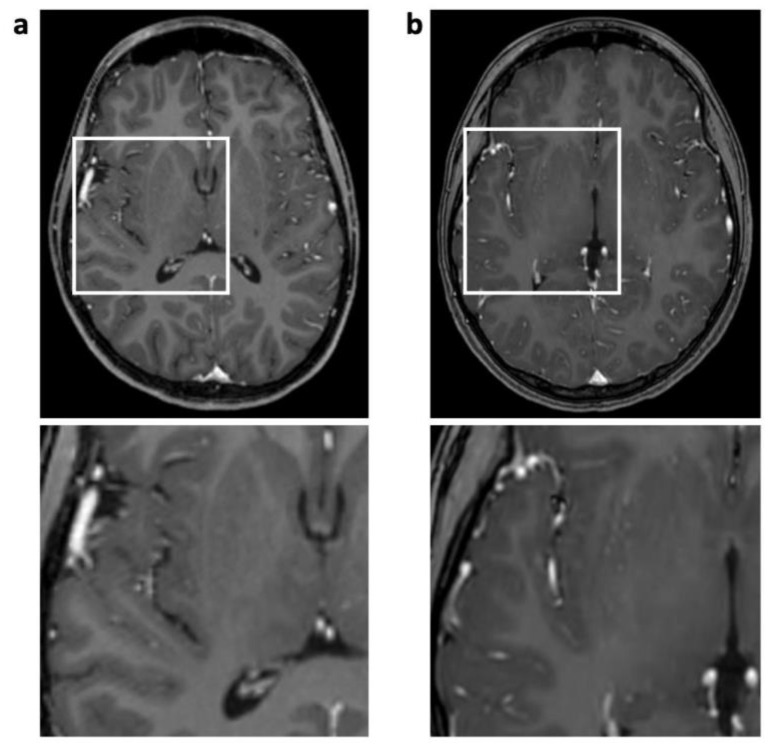
Enhanced 3D T1 TFE images of an 18-year-old male patient with ganglioglioma (not shown). Reconstruction artifact with thin oblique geometrical streaks is seen in SENSE 3D T1 TFE ((**a**), post-surgery); not present in CS 3D T1 TFE (**b**). White box indicates magnification.

A significant decrease in disruptive artifacts was noted for CS 3D T1 TFE pre-contrast (overall *p* < 0.001) and post-contrast (overall *p* < 0.001) images, which is mainly attributable to a reduction in physiological and technical artifacts over the basal ganglia and the cortex. Ghosting and pulsation artifacts of vascular structures were eliminated (*p* = 0.002 for pre-contrast, *p* < 0.001 for post-contrast 3D T1 TFE; see Figure 1), followed by a reduction in grid-like reconstruction artifacts (*p* = 0.008 and *p* = 0.029, respectively; see Figure 2).

**Figure 3:**
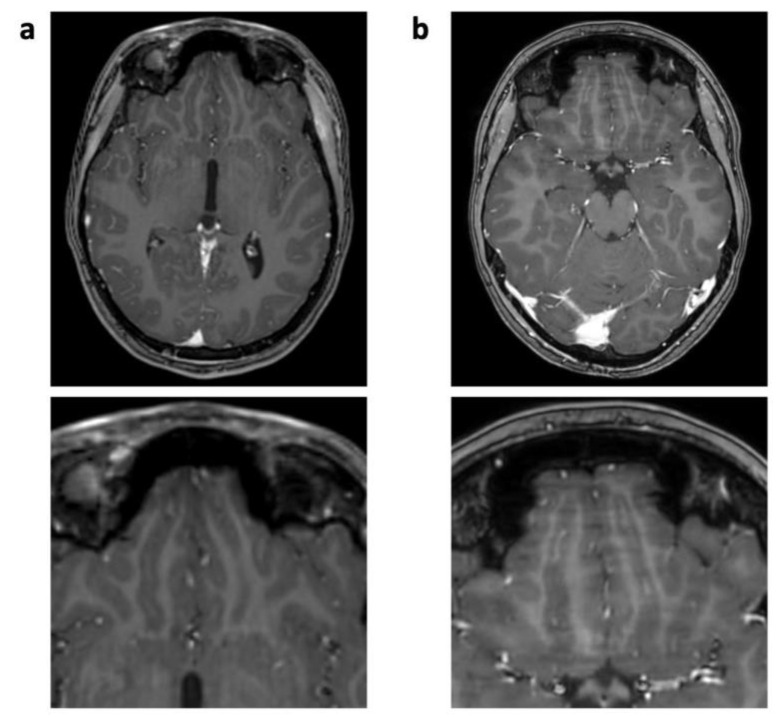
Enhanced 3D T1 TFE images of a 13-year-old female patient with astrocytoma (not shown). “Wavy-lines” artifact with prominent wavy signal distortion over frontal lobes in CS 3D T1 TFE (**b**) was not seen in SENSE 3D T1 TFE (**a**) study prior.

In addition, CS-specific artifacts were noted in both unenhanced and enhanced CS 3D T1 TFE sequences. A “Wavy-lines” artifact occurred in two examinations in CS 3D T1 TFE post-contrast with a broad, wavy pattern of distortion in the horizontal direction over the rostral frontal lobes (Figure 3). Similar but considerably smaller artifacts were seen next to typical susceptibility artifacts caused by a shunt device.

**Figure 4:**
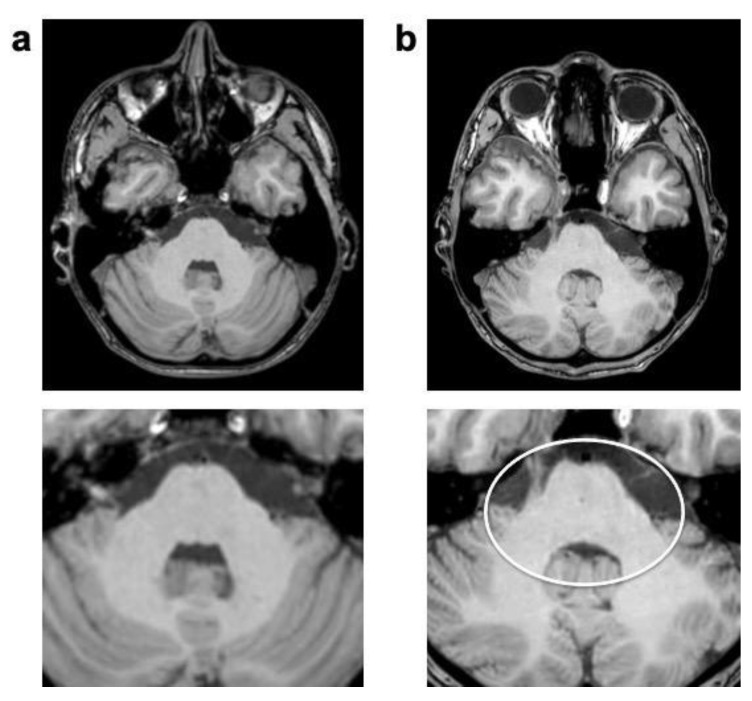
Unenhanced 3D T1 TFE images of a 6-year-old male patient with astrocytoma (not shown). “Starry-sky” artifact presenting as subtle salt-and-pepper-like noisiness in central structures of the acquired volume in CS (**b**); not present in previous SENSE imaging (**a**). White circle indicates artifact.

The “Starry-sky” artifact occurred in all of the unenhanced CS 3D T1 TFE sequences, but only occasionally in the enhanced equivalents. It presented as dotted salt-and-pepper-like noise, mainly at the center of the k-space, but with no preference for specific tissue types or anatomical structures (Figure 4).

**Figure 5:**
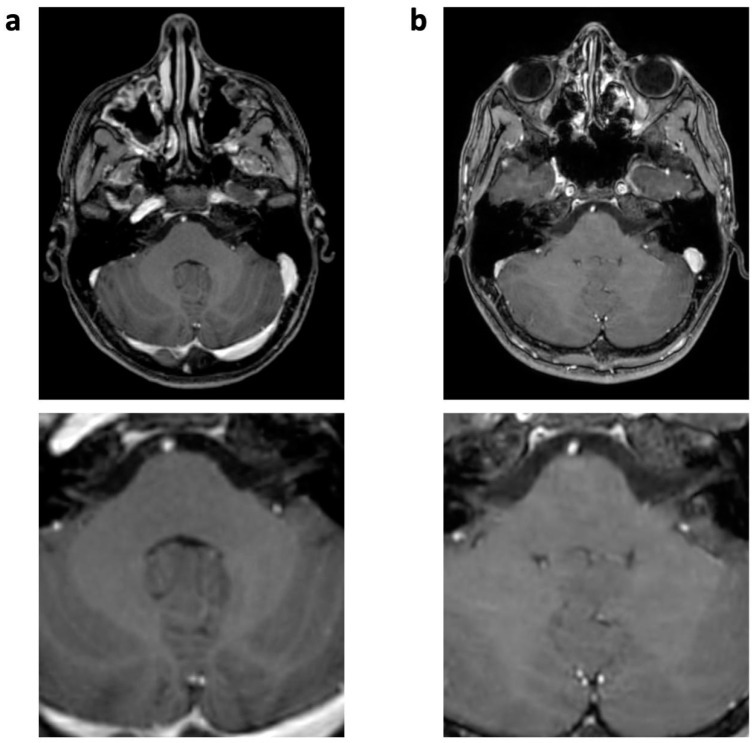
Enhanced 3D T1 TFE images of a 12-year-old male patient with non-germinomatous germ cell tumor (not shown). Wax-layer artifact presenting as patchy to blurred signal inhomogeneity in the pons and cerebellum in CS (**b**); not present in previous SENSE study (**a**).

The “Wax-layer” artifact presented as patchy inhomogeneous blurring of brain structure mainly in post-contrast CS 3D T1 TFE (Figure 5).

**Figure 6:**
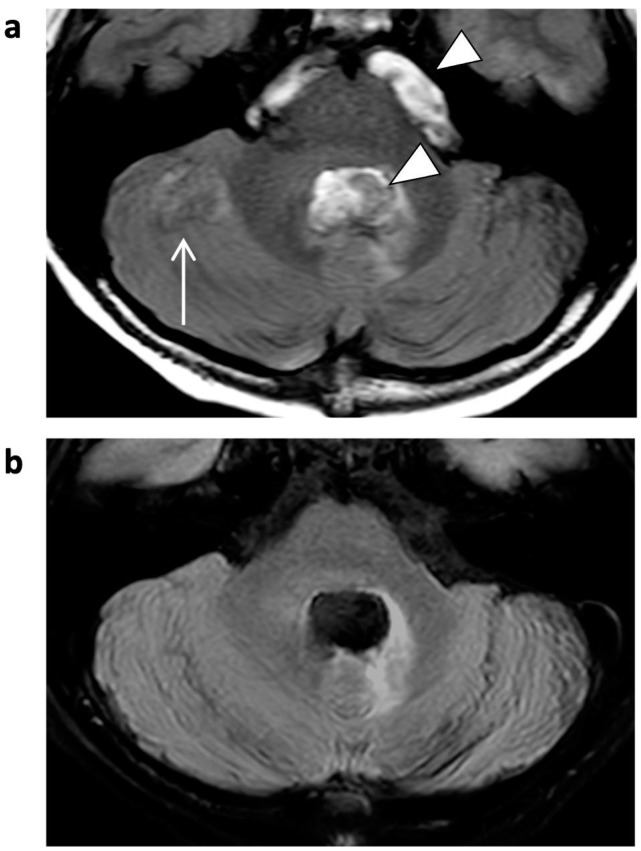
FLAIR images of a 5-year-old male patient with astrocytoma (post-resection). Bright CSF-flow-related enhancement (FRE, arrowheads) in fourth ventricle and prepontine cisterns is seen in SENSE FLAIR (**a**), not present in CS FLAIR (**b**). As a consequence, the CSF-dependent ghosting artifacts (arrow) did not occur.

The CS FLAIR benefited mostly from a reduction in physiological artifacts (overall *p* < 0.001), namely, an improved suppression of cerebro-spinal fluid flow artifacts (*p* < 0.001) and elimination of the dependent ghosting artifacts (*p* < 0.001; see Figure 6). CS FLAIR was the only sequence to demonstrate a significant decrease in motion artifacts (*p* = 0.005) caused by head or eye movement. However, CS FLAIR images were deemed slightly noisier than standard images on visual inspection.

The CS T2 TSE, on the other hand, showed less subjective noising, but the remainder of the artifacts, including CSF-related phenomena, were deemed comparable.

Ringing or truncation artifacts occurred in SENSE and CS 3D T1 TFE and T2 TSE. In CS 3D T1 TFE, ringing became less intense (*p* = 0.010), while it remained comparable in T2 TSE.

No significant differences were noticed for susceptibility effects and chemical shift artifacts between the two groups.

## 4. Discussion

Our study on pediatric brain tumor MR imaging showed that overall artifact burden can be reduced using CS acceleration in comparison to standard parallel imaging acceleration. To the best of our knowledge, the effects of compressed sensing on artifact types and artifact load have not been systematically studied in pediatric brain tumor MR imaging before.

While a number of other studies have described challenges and potential artifacts arising from neuroimaging with 3 Tesla MRI and implementation of compressed sensing and/or SENSE [11,15,23,24], the potential effect of acceleration techniques on artifact appearance in pediatric MR imaging protocols has only been investigated to a limited extent and mainly with regards to abdominal imaging [9,25].

MR brain tumor imaging relies on the best possible image quality in order to maximize diagnostic confidence, but pediatric neuroimaging is often challenging in patients with small body volumes. Acceleration techniques that maintain or even improve image quality are therefore highly desired [14]. Also, pathologic findings often are of millimeter size and can be found in areas which are frequently altered by artifacts, e.g., in periventricular localization, adjacent to surgical sites, or next to surgical material and shunt devices [26,27]. Thus, the appearance of artifacts in these particular areas has the potential to affect diagnostic confidence.

Some neuro-oncologic patients might show limited compliance due to their altered state of consciousness, or physical impairment caused by the primary disease or treatment, resulting in motion artifacts, as patients are not able to keep their head still for a long period of time. The same problem is seen in young children, who often are anxious or bored during an MR examination, and in sedated children who present with uncontrolled movement of head or limbs. This challenge in oncologic and pediatric MR imaging can be addressed by the choice of movement-robust sequences and a reduction of scan time; however, these effects might be observed best in examination protocols with longer duration. In our study, a significant reduction in motion artifacts was seen in the CS FLAIR sequence, which was shortened most significantly by compressed sensing implementation [13].

Especially younger pediatric patients often demonstrate pronounced CSF flow artifacts. Their CSF circulation can differ from that of adult patients as it is affected by physiological parameters such as respiratory rate, arterial pulsation, and blood pressure [28,29]. Ghosting of these artifacts, as frequently seen in the posterior fossa, heavily disguises the detectability of local pathologic findings. In children, pathologic findings in the posterior fossa also occur often due to the statistically high likelihood of pediatric primary CNS tumors originating around the fourth ventricle.

With adequate suppression of the CSF signal by reduced TR and TI, such ghosting artifacts and signal loss [17] that occurred at basal cisterns, the third ventricle, and the foramen of Monro were dramatically reduced in the CS FLAIR sequence, whereas there was no apparent difference in T2 sequences under comparable parameter settings.

Interestingly, in 3D T1 gradient echo sequences, ghosting artifacts not related to CSF flow but to pulsation of the arteries of the circle of Willis were also eliminated in the CS protocol. This can be explained by the incoherent sampling pattern used in compressed sensing instead of the regular periodic undersampling in conventional SENSE [4,30,31]. The decrease in reconstruction artifacts in CS 3D T1 TFE sequences might be caused by the CS-specific L1 reconstruction algorithm in combination with the incoherent sampling pattern, which is designed to minimize disruptive signals.

A higher spatial resolution in CS 3D T1 TFE also contributed to a reduction in ringing artifacts that occurred at anatomical borders where signal intensity changed abruptly. The significant decrease in ringing artifacts in CS FLAIR may be due to better fat suppression.

The technical foundation of the CS 3D T1 TFE sequence serves as a potential explanation for CS-specific artifacts as well. Again, the mathematical random varying density undersampling scheme in CS could explain the frequently occurring “Starry-sky” artifact, as the center of the k-space might be too sparsely represented, resulting in too few coefficients during the mathematical iterative image reconstruction process [30,32]. Its strength of occurrence showed no correlation with the field of view or head volume, as it was observed in examinations of all patients with different body sizes. Although the “Starry-sky” artifact was found to be only slightly disruptive and therefore not deemed diagnostically impactful, further careful adjustment of the CS factor in accordance with the SNR might help to reduce the strength of this artifact. A potential cause of the wax-layer artifact could be a strong denoising level, where large sparsity in general is assumed in the algorithm. Still, as the CS denoising settings remained unchanged for all patients over the period of the study, and the artifact appeared only sporadically within our population, subtle patient motion could also have caused this particular artifact, as it typically creates blurring or smearing in compressed sensing imaging. The “Wavy-lines” artifact’s close anatomical relation to the air-filled paranasal sinuses and shunt devices indicates a correlation with larger gradients between different types of tissue, contributing to field inhomogeneity. Although Sartoretti et al. described a strong correlation between a similar streaky linear artifact and having a smaller reconstruction voxel size than acquisition voxel size [15], the “Wavy-lines” artifact does not appear to be caused by this, as voxel sizes remained comparable during our study.

The balance between image quality and noise depends on coil sensitivity and the acceleration factor [32,33,34]. With regards to subjective noisiness, it aims for the most beneficial compromise during the compressed sensing implementation process, with the aim of optimizing general image quality and examination time for overall protocol improvement [13]. As quantitative noise evaluation did not show significant differences between the two protocols but the subjective noisiness of T2 TSE and FLAIR sequences differed, there is still space for further adjustment of the denoising factor, acceleration factor, and TR.

There were limitations to our study that need to be outlined. The small study cohort with *n* = 22 patients might not cover the full extent of potential artifacts in brain MRI. Total blinding of protocols was not possible due to the distinct image impression of conventional parallel imaging and CS usage, which could easily be identified by an experienced reader. Additional adjustments of the CS FLAIR sequence parameters regarding CSF suppression might disguise the effects of CS on CSF artifact appearance; however, these amendments were deemed necessary in the context of compressed sensing implementation in order to achieve superior image quality [13]. Prior to the study, an optimization of sequences was conducted during a pilot phase based on the previous experience of other centers and the recent literature [3,5,9,12,22,35,36,37,38,39].

## 5. Conclusions

In conclusion, CS contributes to a reduction in overall artifact burden and even the elimination of certain physiology-related artifacts in dedicated pediatric brain tumor MRI. However, to a lesser extent, the introduction of CS can also add new artifacts. Readers not familiar with CS therefore need to become accustomed to CS-specific artifacts to avoid pitfalls in interpretation. The artifact burden observed while utilizing iterative reconstruction algorithms should be monitored and regularly addressed during the optimization process. Future studies are needed to further investigate the artifact impact on diagnostic performance.

## Figures and Tables

**Table 1:** Comparison of sequence data for the SENSE and compressed sensing (CS) pediatric brain tumor protocols.

	3D T1 TFE		T2 TSE		FLAIR	
	SENSE	CS	SENSE	CS	SENSE	CS
Scan time (min:sec)	03:38	03:00	03:36	02:07	03:51	02:38
Acceleration	SENSE 1.2 × 2.2	3.3	-	1.3	SENSE 1.8 × 1.3	4.5
TR/TE (ms)	8.3/3.8	8.6/4.0	3000/80	3954/80	11,000/125	4800/396
TI delay (ms)	956.8	989.9	-	-	2800	1650
SNR^a^ (arbitrary)	167.0	145.7	155.3	189.3	205.3	222.3
FOV (mm^3^)	240 × 240 × 175	240 × 240 × 175	230 × 182 × 152	230 × 182 × 152	230 × 183 × 138	230 × 179 × 152
Voxel size [ACQ] (mm^3^)	1.0 × 1.0 × 1.0	0.85 × 0.85 × 0.85	0.55 × 0.65 × 3.0	0.55 × 0.65 × 3.0	0.65 × 0.87 × 3.0	0.75 × 0.75 × 3.3
Voxel size [REC] (mm^3^)	0.9 × 0.9 × 1.0	0.43 × 0.43 × 0.43	0.4 × 0.4 × 3.0	0.4 × 0.4 × 3.0	0.34 × 0.34 × 3.0	0.34 × 0.34 × 3.3

SENSE sensitivity encoding, CS compressed sensing sensitivity encoding, 3D three-dimensional, TFE turbo field echo, TSE turbo spin echo, FLAIR fluid-attenuated inversion recovery, TR repetition time, TE echo time, TI inversion time, SNR signal-to-noise ratio, FOV field of view, ACQ voxel acquisition voxel size, REC voxel reconstruction voxel size. SNR^a^ (in arbitrary units) measurements were conducted in a standard phantom with separate noise maps (for details, see text).

**Table 2:** Evaluation of artifact occurrence and strength in 3D T1 TFE.

Artifact Category	Type of Artifact	SENSE		Compressed Sensing (CS)		*p*
		Scans Affected *n* (%)	Artifact Strength Sum Score (Mean ± SD)	Scans Affected *n* (%)	Artifact Strength Sum Score (Mean ± SD)	
Physiology-related	Motion	2 (9%)	2 (0.09 ± 0.29)	0	0	0.180
	Ringing	11 (50%)	14 (0.64 ± 0.73)	10 (45%)	10 (0.45 ± 0.61)	0.010
	CSF flow	0	0	0	0	0
	Pulsation/ghosting	12 (55%)	15 (0.68 ± 0.72)	0	0	0.002
Physics-related	Chemical shift	22 (100%)	30 (1.36 ± 0.49)	22 (100%)	36 (1.64 ± 0.49)	0.030
	Susceptibility effects	21 (95%)	35 (1.59 ± 0.59)	21 (95%)	34 (1.55 ± 0.60)	0.285
Technique-related	Straight bands	10 (45%)	10 (0.45 ± 0.51)	1 (5%)	1 (0.05 ± 0.21)	0.008
	Starry sky	0	0	22 (100%)	33 (1.50 ± 0.51)	
	Wax layer	0	0	1 (5%)	1 (0.05 ± 0.21)	
	Wavy lines	0	0	1 (5%)	1 (0.05 ± 0.21)	
Overall			4.82 ± 1.50		3.68 ± 1.04 *	<0.001

SENSE sensitivity encoding, CS compressed sensing sensitivity encoding, 3D three-dimensional, TFE turbo field echo, CSF cerebro-spinal fluid. Scans affected are given as absolute number of scans (n) and percentage of scans. Artifact strength given as numeric score summarizing all 22 scans with mean ± SD (artifact strength 0–2 points per scan; maximum artifact strength sum score per sequence and artifact 44 points; see Table S2). The mean overall score summarizes the artifact burden from all artifact types’ strength scores with values given as mean ± SD. * The CS-specific artifacts are not included in the mean overall score as they did not occur in the SENSE protocol.

**Table 3:** Evaluation of artifact occurrence and strength in 3D T1 TFE post-contrast.

Artifact Category	Type of Artifact	SENSE		Compressed Sensing (CS)		*p*
		Scans Affected *n* (%)	Artifact Strength Sum Score (Mean ± SD)	Scans Affected *n* (%)	Artifact Strength Sum Score (Mean ± SD)	
Physiology-related	Motion	4 (18%)	6 (0.27 ± 0.63)	1 (5%)	1 (0.05 ± 0.21)	0.066
	Ringing	14 (64%)	19 (0.64 ± 0.73)	11 (55%)	14 (0.45 ± 0.51)	0.060
	CSF flow	0	0	1 (5%)	2 (0.09 ± 0.43)	0.317
	Pulsation/ghosting	17 (77%)	23 (1.05 ± 0.72)	0	0	<0.001
Physics-related	Chemical shift	22 (100%)	31 (1.41 ± 0.50)	22 (100%)	31 (1.41 ± 0.50)	0.354
	Susceptibility effects	21 (95%)	37 (1.68 ± 0.57)	21 (95%)	35 (1.59 ± 0.59)	0.180
Technique-related	Straight bands	9 (41%)	10 (0.45 ± 0.60)	2 (9%)	2 (0.09 ± 0.29)	0.029
	Starry sky	0	0	12 (55%)	14 (0.45 ± 0.51)	
	Wax layer	0	0	11 (50%)	11 (0.50 ± 0.51)	
	Wavy lines	0	0	2 (9%)	2 (0.09 ± 0.43)	
Overall			5.73 ± 1.72		3.86 ± 1.21 *	<0.001

SENSE sensitivity encoding, CS compressed sensing sensitivity encoding, 3D three-dimensional, TFE turbo field echo, CSF cerebro-spinal fluid. Scans affected are given as absolute number of scans (n) and percentage of scans. Artifact strength given as numeric score summarizing all 22 scans with mean ± SD (artifact strength 0–2 points per scan; maximum artifact strength sum score per sequence and artifact 44 points; see Table S2). The mean overall score summarizes the artifact burden from all artifact types’ strength scores with values given as mean ± SD. * The CS-specific artifacts are not included in the mean overall score as they did not occur in the SENSE protocol.

**Table 4:** Evaluation of artifact occurrence and strength in T2 TSE.

Artifact Category	Type of Artifact	SENSE		Compressed Sensing (CS)		*p*
		Scans Affected *n* (%)	Artifact Strength Sum Score (Mean ± SD)	Scans Affected *n* (%)	Artifact Strength Sum Score (Mean ± SD)	
Physiology-related	Motion	3 (14%)	4 (0.18 ± 0.50)	3 (14%)	3 (0.14 ± 0.35)	0.423
	Ringing	9 (41%)	12 (0.55 ± 0.74)	10 (45%)	12 (0.55 ± 0.67)	0.192
	CSF flow	22 (100%)	38 (1.73 ± 0.46)	22 (100%)	41 (1.86 ± 0.35)	0.080
	Pulsation/ghosting	17 (77%	22 (1.00 ± 0.69)	20 (91%)	26 (1.18 ± 0.59)	0.041
Physics-related	Chemical shift	1 (5%)	1 (0.05 ± 0.21)	1 (5%)	1 (0.05 ± 0.21)	0
	Susceptibility effects	19 (86%)	23 (1.05 ± 0.58)	19 (86%)	23 (1.05 ± 0.58)	0
Technique-related	Straight bands	0	0	0	0	
	Starry sky	0	0	0	0	
	Wax layer	0	0	0	0	
	Wavy lines	0	0	0	0	
Overall			4.55 ± 1.53		4.82 ± 1.10 *	0.018

SENSE sensitivity encoding, CS compressed sensing sensitivity encoding, 3D three-dimensional, TFE turbo field echo, CSF cerebro-spinal fluid. Scans affected are given as absolute number of scans (n) and percentage of scans. Artifact strength given as numeric score summarizing all 22 scans with mean ± SD (artifact strength 0–2 points per scan; maximum artifact strength sum score per sequence and artifact 44 points; see Table S2). The mean overall score summarizes the artifact burden from all artifact types’ strength scores with values given as mean ± SD. * The CS-specific artifacts are not included in the mean overall score as they did not occur in the SENSE protocol.

**Table 5:** Evaluation of artifact occurrence and strength in FLAIR.

Artifact Category	Type of Artifact	SENSE		Compressed Sensing (CS)		*p*
		Scans Affected *n* (%)	Artifact Strength Sum Score (Mean ± SD)	Scans Affected *n* (%)	Artifact Strength Sum Score (Mean ± SD)	
Physiology-related	Motion	10 (45%)	11 (0.50 ± 0.60)	0	0	0.005
	Ringing	21 (95%)	24 (1.09 ± 0.43)	6 (27%)	6 (0.27 ± 0.46)	<0.001
	CSF flow	22 (100%)	43 (1.95 ± 0.21)	8 (36%)	8 (0.36 ± 0.49)	<0.001
	Pulsation/ghosting	18 (82%)	30 (1.36 ± 0.79)	0	0	<0.001
Physics-related	Chemical shift	6 (27%)	(0.27 ± 0.46)	0	0	0.028
	Susceptibility effects	19 (86%)	20 (0.91 ± 0.43)	19 (86%)	23 (1.05 ± 0.58)	0.109
Technique-related	Straight bands	0	0	0	0	
	Starry sky	0	0	0	0	
	Wax layer	0	0	0	0	
	Wavy lines	0	0	0	0	
Overall			6.09 ± 1.72		1.68 ± 0.72 *	<0.001

SENSE sensitivity encoding, CS compressed sensing sensitivity encoding, 3D three-dimensional, TFE turbo field echo, CSF cerebro-spinal fluid. Scans affected are given as absolute number of scans (n) and percentage of scans. Artifact strength given as numeric score summarizing all 22 scans with mean ± SD (artifact strength 0–2 points per scan; maximum artifact strength sum score per sequence and artifact 44 points; see Table S2). The mean overall score summarizes the artifact burden from all artifact types’ strength scores with values given as mean ± SD. * The CS-specific artifacts are not included in the mean overall score as they did not occur in the SENSE protocol.

## Data Availability

Data are available on request.

## Supplementary Materials

The following supporting information can be downloaded at: https://www.mdpi.com/article/10.3390/jcm12175732/s1, Table S1: Patient demographics; Table S2: Description of artifacts assessed in this study.
