# Supplementary material for: Evaluation of Artifact Appearance and Burden in Pediatric Brain Tumor MR Imaging with Compressed Sensing in Comparison to Conventional Parallel Imaging Acceleration
Source: J Clin Med. 2023 Sep 3;12(17):5732. doi: 10.3390/jcm12175732 (PMC10489124; doi:10.3390/jcm12175732)
Supplement: Supplementary file 1 [file jcm-12-05732-s001.zip › jcm-2566733-supplementary.pdf]

Journal of
Clinical Medicine

Evaluation of Artifact Appearance and Burden in Pediatric Brain Tumor MR Imaging with Compressed Sensing in Comparison to Conventional Parallel Imaging Acceleration

Supplementary Materials: Table S1. Patient Demographics; Table S2. Description of artifacts assessed in this study.

Table S1. Patient Demographics.

Characteristics	Values
No. Patients	22
Sex, male:female	15:7
Age, mean ± 1 SD (range), years	10.4 ± 4.6 (2.3-18.9)
male	10.4 ± 5.0 (2.3-18.9)
female	10.3 ± 4.1 (4.3-14.7)
General Anesthesia, No. Patients	5
Age, mean ± 1 SD (range), years	4.7 ± 1.5 (2.3-5.9)
Underlying malignancy, No. Patients (%)	
Astrocytoma	8 (36.4%)
Medulloblastoma	4 (18.2%)
Ependymoma	3 (14.6%)
*other primary CNS **	5 (22.7%)
*other ***	2 (9.1%)
Clinical Staging, stable:not stable	12:10
Time since diagnosis, mean ± 1 SD (range), months	
long-term:short-term	18:4
long-term	38.9 ± 22.6 (13-100)
short-term	7.8 ± 2.2 (5-10)
Therapeutic Modality, No. Patients (%)	
Tumor resection	17 (77.3%)
Chemotherapy	15 (68.2%)
Radiation therapy	11 (50.0%)

* Other primary CNS tumors: Non-germinomatous germ cell tumor NGGCT (n=2), Atypical teratoid/rhabdoid tumor AT/RT, Peripheral (a)melanotic neuroectodermal tumor of infancy PMNT, Ganglioglioma.
** other: Teratoma, Haemangiopericytoma.

Table S2. Description of artifacts assessed in this study.

Artifact Category	Type of Artifact	Appearance
Physiology-related	Motion	Image blurring with aliasing or repetitive structures[1]
	Ringing	Bright or dark parallel lines to edges of abrupt signal intensity changes[1]
	CSF Flow	Hyperintense fluid signal on FLAIR images (FRE), and hypointense on T2 images (TOF loss), with linear or amorph configuration depending on direction of CSF flow (straight or turbulent)[2]
	Pulsation/Ghosting	periodic replica of a pulsatile structure at abnormal position[1]
Physics-related	Chemical Shift	spatial misregistration around fatty and watery tissues[1]
	Susceptibility effects	Local signal void due to susceptibility differences[1]
Technique-related	Straight bands	Multiple grid-like thick or thin lines, similar to spike noise/data error artifact[3]
	Starry-sky	Dotted salt-and-pepper-like noisiness mainly at the center of the k-space[4]
	Wax-layer	Patchy inhomogeneity of brain structure with decrease of sharpness[4]
	Wavy-lines	Areas of broad, wavy distortion

CSF cerebro-spinal fluid, *CS* compressed sensing sensitivity encoding.

1 Heiland, S. From A as in Aliasing to Z as in Zipper: Artifacts in MRI. *Clin Neuroradiol* 18, 25–36 (2008). https://doi.org/10.1007/s00062-008-8003-y

2 Lisanti C, Carlin C, Banks KP, Wang D. Normal MRI appearance and motion-related phenomena of CSF. AJR Am J Roentgenol. 2007 Mar;188(3):716-25. doi: 10.2214/AJR.05.0003. PMID: 17312059.

3 Zhuo J, Gullapalli RP. AAPM/RSNA physics tutorial for residents: MR artifacts, safety, and quality control. Radiographics. 2006 Jan-Feb;26(1):275-97. doi: 10.1148/rg.261055134. PMID: 16418258.

4 Sartoretti T, Reischauer C, Sartoretti E, Binkert C, Najafi A, Sartoretti-Schefer S. Common artefacts encountered on images acquired with combined compressed sensing and SENSE. Insights Imaging. 2018 Dec;9(6):1107-1115. doi: 10.1007/s13244-018-0668-4. Epub 2018 Nov 8. PMID: 30411279; PMCID: PMC6269339.
